# Compressed sensing accelerated 4D-flow MRI in the murine aorta

**DOI:** 10.1186/1532-429X-15-S1-W37

**Published:** 2013-01-30

**Authors:** J Fluckiger, BD Allen, K Caldock, M Markl, JE Schneider

**Affiliations:** 1 Cardiovascular Medicine, University of Oxford, Oxford, UK; 2 Radiology & Biomedical Engineering, Northwestern University, Chicago, IL, USA

## Background

A key determinant of cardiac pump function is the complex interplay between the heart and the arterial system, (i.e. arterio-ventricular coupling). Aging and pathological changes such as atherosclerosis alter elastic properties of the vessel wall, and therefore adversely influence hemodynamic parameters. Mice are commonly used to study cardiovascular disease, but very little has been published on comprehensive blood flow measurements in these models. We therefore sought to establish a compressed sensing (CS) accelerated, phase contrast MR technique (4D flow MRI) for measuring aortic 3D hemodynamics in the mouse.

## Methods

The experiments were carried out on female C57Bl6 mice (28.0 ± 0.1g, n= 3) using a quadrature driven birdcage resonator (id 33mm) on a 9.4T Agilent MR system. A four-point referenced, multi-frame phase contrast MR sequence (TE/TR=1.86/4.6ms, 128x128, FOV 25.6x25.6mm, 0.3mm slice thickness, 12-16 contiguous slices, venc_x/y/z_=150cm/s, 2 averages) was used to encode blood flow in the aorta in 3D + time (i.e. 4D). To investigate whether or not CS can be used to accelerate the experiments, up to 3-fold undersampling and subsequent CS-reconstruction was performed in post-processing as reported previously (Wech, T., et al., J Magn Reson Imaging, 2011. 34(5): p. 1072-9). All image sets were processed using in-house software for noise reduction, eddy current correction, and anti-aliasing. 3D blood flow visualization (systolic 3D stream lines) and quantification (peak flow in retrospectively positioned analysis planes in the ascending and descending aorta) was performed using EnSight (CEI, Apex, NC).

## Results

**Figure 1:**
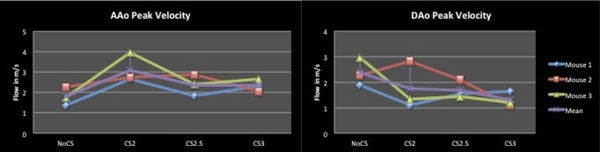


Figure 1 shows 3D streamline visualization of peak systolic blood flow in the aorta for fully-sampled, 2-, 2.5- and 3-fold undersampled CS-reconstructed data, respectively, illustrating that the principle flow distribution is preserved in the undersampled data. Figure 2 depicts the individual and the average peak velocities in both the ascending (AAo) and the descending (DAo) aorta for the different data sets suggesting a trend towards higher / lower peak velocities in the AAo / Dao in the undersampled data.

## Conclusions

This is the first study to combine CS with phase-contrast MRI to quantify 4D-blood flow in the mouse aorta. While the initial data indicate that at least a 2.5-fold reduction in scan time should be possible, more work will be required to better characterise and to quantify the impact CS on the measured blood flow parameters.

## Funding

This work was funded by the British Heart Foundation (BHF).

